# Pharmacotherapy for Co-Occurring Alcohol Use Disorder and Post-Traumatic Stress Disorder: Targeting the Opioidergic, Noradrenergic, Serotonergic, and GABAergic/Glutamatergic Systems

**DOI:** 10.35946/arcr.v39.2.09

**Published:** 2018

**Authors:** Terril L. Verplaetse, Sherry A. McKee, Ismene L. Petrakis

**Affiliations:** Terril L. Verplaetse, Ph.D., is an associate research scientist in the Department of Psychiatry, Yale School of Medicine, New Haven, Connecticut. Sherry A. McKee, Ph.D., is a professor of psychiatry at the Yale School of Medicine, New Haven, Connecticut. Ismene L. Petrakis, M.D., is a professor of psychiatry at the Yale School of Medicine, New Haven, Connecticut

**Keywords:** alcohol, alcohol use disorder (AUD), comorbidity, pharmacotherapy, post-traumatic stress, post-traumatic stress disorder (PTSD)

## Abstract

Alcohol use disorder (AUD) and post-traumatic stress disorder (PTSD) are highly comorbid, and treatment outcomes are worse in individuals with both disorders. Several neurobiological systems have been implicated in the development and maintenance of AUD and PTSD, and pharmacologic interventions targeting these systems for singular diagnoses of AUD or PTSD have proven effective. However, there are no established treatments for co-occurring AUD and PTSD, and relatively few studies have examined potential pharmacotherapy for treating symptoms of both AUD and PTSD in comorbid populations. This review provides a brief overview of the studies to date on pharmacotherapeutic treatment interventions for comorbid AUD and PTSD and highlights future directions for promising targets that have potential in the treatment of individuals with this dual diagnosis. Clinical implications of these findings are also discussed. While current medications targeting the opioidergic, noradrenergic, serotonergic, and GABAergic/glutamatergic brain systems are only modestly efficacious in improving symptoms in individuals with comorbid AUD and PTSD, novel targets within these neurobiological systems may be clinically useful for treating alcohol use outcomes and PTSD symptom severity. More work is needed to optimize pharmacologic treatment strategies that target both alcohol-motivated behavior and PTSD-related symptoms in individuals with co-occurring AUD and PTSD.

## Introduction

Over the past decade, 12-month alcohol use, high-risk drinking, and alcohol use disorder (AUD) have increased by 11.2%, 29.9%, and 49.4%, respectively, in the United States.^1^ In addition to increasingly high prevalence rates of AUD and the severe health and economic consequences associated with the disorder,^2^ AUD is also highly comorbid with other psychiatric illnesses. One such comorbidity is post-traumatic stress disorder (PTSD). PTSD is a chronic and disabling disorder and is characterized by intrusive or distressing thoughts, persistent avoidance of stimuli related to the traumatic event, negative alterations in cognition or mood, and symptoms of arousal following exposure to a traumatic event. Lifetime and 12-month prevalence of PTSD in the general population are 6.1% and 4.7%, respectively.^3^ This percentage is larger in certain populations, such as veteran populations, where lifetime prevalence ranges from 6.9% in U.S. veterans to 37.3% in war-specific cohorts.^4^ Previous estimates suggest that individuals with PTSD are more likely to have comorbid AUD, as much as 42% of individuals within the general population^5^ and 55% of veterans.^4^ This is consistent with recent epidemiologic findings demonstrating a reciprocal relationship between the two disorders, such that the odds of having PTSD are significantly greater in individuals with lifetime AUD.^6^

Individuals with both AUD and PTSD typically exhibit worse outcomes, ranging from social consequences and psychological problems to treatment responses, when compared with individuals with either diagnosis alone.^7^ Individuals with comorbid AUD and PTSD tend to have more severe PTSD symptoms, increased alcohol-related problems, increased risk of relapse, more frequent hospitalizations, increased emotional dysregulation, and increased odds of additional psychiatric comorbidity and suicide attempts than individuals with either disorder alone.^8^,^9^ Other difficulties in this comorbid population include increased unemployment and homelessness. To further complicate the picture, only 19.8% and 59.4% of those with singular diagnoses of lifetime AUD and PTSD, respectively, ever seek or receive treatment,^3^,^6^ and treatment-seeking rates in individuals with comorbid AUD and PTSD are very low.^8^ Treatment adherence and response are also poorer in individuals with both disorders, compared with individuals with a singular diagnosis.^9^

The neurobiology underlying AUD and PTSD is complex and not fully understood. While not comprehensive of all systems, the opioid, norepinephrine, serotonin, gamma-aminobutyric acid (GABA), and glutamate neurotransmitter systems are independently implicated in the pathophysiology of the development and maintenance of both AUD and PTSD.^9^,^10^ Extensive research has focused on the opioidergic system specifically for AUD^11^ and to a lesser extent for PTSD.^12^ More recent attention has focused on the importance of the role of brain stress systems in both drinking behavior^13^ and PTSD symptomology,^14^ highlighting the importance of the noradrenergic system. “Feed-forward” mechanisms within the stress systems may mediate exaggerated stress responses in individuals with AUD and PTSD. In brief, corticotropin-releasing hormone stimulates the release of norepinephrine in response to stress.^15^ Increased levels of norepinephrine are thought to play an important role in arousal, drug-motivated behaviors, withdrawal, and PTSD. Further, norepinephrine release and stress can lead to the release of serotonin,^15^ which is commonly associated with anxiety disorders and depression but also PTSD. Recent evidence suggests that GABAergic and glutamatergic pathways may also be linked to AUD and PTSD. GABA and glutamate work synergistically and are important in neural plasticity, memory consolidation, fear learning, anxiety, and drug craving,^16^ lending support for these systems in the maintenance of AUD and PTSD. Targeting alcohol responses and stress reactivity within these systems to treat comorbid AUD and PTSD represents a niche area of research and warrants further investigation.

Although several thorough reviews on interventions for comorbid AUD and PTSD have been published recently,^16^ this review aims to discuss pharmacotherapy for comorbid AUD and PTSD in terms of five neurobiological systems: the opioidergic, noradrenergic, serotonergic, GABAergic, and glutamatergic systems. While not comprehensive of all systems that may be dysregulated by both AUD and PTSD, most of the existing work examining pharmacologic treatments in individuals with comorbid AUD and PTSD have focused on these neurobiological systems. To date, there are 12 studies, including randomized controlled trials, small open-label trials, and retrospective studies, that have examined pharmacotherapy targeting opioidergic, noradrenergic, serotonergic, and GABAergic/ glutamatergic systems for the treatment of co-occurring AUD and PTSD. These studies, reviewed in this article, indicate that there is limited to modest efficacy in reducing both alcohol use outcomes and symptoms associated with PTSD in individuals with a dual diagnosis. Because effective pharmacologic treatments remain elusive, finding novel treatment targets or pharmacotherapeutic treatment strategies for comorbid AUD and PTSD is critical.

The purpose of this review is to provide an overview of current clinical trials and human experimental studies examining pharmacotherapy for comorbid AUD and PTSD. For each neurobiological system discussed, we provide potential candidates that could be examined in future studies on effective treatment targets. Finally, we provide future research directions and suggestions that have potential to advance the field toward improvements in clinical treatment options for individuals with both AUD and PTSD. While there is a rich literature on behavioral treatments for comorbid AUD and PTSD, behavioral interventions are beyond the scope of the present review (see Simpson, Lehavot, and Petrakis for a review of behavioral clinical trials).^17^

## Agents Acting on the Opioidergic System

Naltrexone, a nonselective opioid antagonist that is one of four U.S. Food and Drug Administration (FDA)-approved medications to treat AUD, was approved based on two randomized controlled trials that demonstrated reductions in alcohol craving, drinking days, and risk to alcohol relapse.^10^ Few studies have examined naltrexone for PTSD without comorbidity, and results are mixed and limited by small sample sizes.^12^ To date, three studies have examined oral naltrexone for treating co-occurring AUD and PTSD,^18^–^20^ demonstrating modest efficacy on alcohol use outcomes and craving and limited efficacy for improving some PTSD symptoms. In veterans with comorbid AUD and PTSD, naltrexone, when compared with placebo, effectively reduced the percentage of heavy-drinking days and increased consecutive days of abstinence.^18^ But in a separate study of veterans with comorbid AUD and PTSD, naltrexone given in addition to paroxetine or desipramine, serotonin and norepinephrine reuptake inhibitors, respectively, decreased alcohol craving but did not influence drinking outcomes.^19^ Both studies used 50 mg/day naltrexone, and the latter study did not examine naltrexone alone.

One other study examined 100 mg/day naltrexone in both civilians and veterans with comorbid AUD and PTSD.^20^ In that study, naltrexone, relative to placebo, decreased alcohol craving and the percentage of drinking days. PTSD symptom severity declined over the course of all three studies, but there was no advantage of naltrexone over placebo. Further, in an exploratory analysis, Foa and colleagues demonstrated that individuals treated with naltrexone and prolonged exposure therapy were more likely to have a clinically meaningful reduction in PTSD symptom severity at 6-month follow-up, compared with the other three treatment conditions: placebo plus prolonged exposure therapy, naltrexone plus supportive counseling, or placebo plus supportive counseling.^20^ It should be noted that these studies were conducted with veterans and civilians who had a dual diagnosis of AUD and PTSD, suggesting efficacy across multiple populations.

## Other Opioidergic Medications

Naltrexone was efficacious in reducing alcohol use outcomes but did not consistently or robustly improve PTSD symptoms in individuals with AUD and PTSD. Other medications targeting the opioidergic system show promise in reducing symptoms associated with singular diagnoses of AUD or PTSD, but these medications have yet to be tested in individuals with AUD and PTSD comorbidity. For alcohol, randomized controlled trials demonstrate that nalmefene, a combined mu-opioid receptor antagonist and partial kappa-opioid receptor agonist, is effective in reducing a number of alcohol use outcomes, compared with placebo, in individuals with AUD (see Mann et al. for a review).^21^ Older studies have also evaluated nalmefene for PTSD, with some indication that nalmefene reduces emotional numbing, nightmares, flashbacks, intrusive thoughts, and other PTSD-associated symptoms.^22^ However, to date, no studies have examined nalmefene for comorbid AUD and PTSD.

Other findings suggest that signaling at primarily kappa-opioid receptors plays a role in alcohol-motivated behaviors. Preclinical studies suggest that the kappa-opioid receptor antagonists JDTic and nor-binaltorphimine (nor-BNI) attenuate alcohol self-administration and cue-induced reinstatement of alcohol-seeking in rodents, with some indication that kappa-opioid receptor antagonists are more effective in alcohol-dependent versus nondependent animals.^23^ Kappa-opioid receptors are also thought to play a role in regulating stress and anxiety, and they have been suggested for use as pharmacologic agents for the treatment of stress-related psychiatric disorders.^24^ Because kappa-opioid receptor antagonists have the ability to reduce persistent hyperarousal and improve alterations in cognition, characteristic symptoms of PTSD, they may be useful for this clinical indication. Unfortunately, not many studies have examined these pharmacologic treatments for AUD or PTSD alone or for their comorbidity. Targeting kappa-opioid receptors may be a promising avenue for individuals with AUD and PTSD, especially for individuals with severe AUD, as JDTic was more effective in alcohol-dependent rodents than in nondependent rodents.

## Agents Acting on the Noradrenergic System

Prior studies evaluating the efficacy of prazosin, an alpha_1_-adrenergic antagonist, for separate indications of AUD^25^,^26^ and PTSD^27^ have demonstrated promising results in reducing alcohol- and PTSD-related outcomes, respectively. However, to date, only two studies have evaluated prazosin for co-occurring AUD and PTSD, with mixed results. In the first study, a 6-week, placebo-controlled trial of 16 mg/day of prazosin was effective in reducing percent drinking days per week and percent heavy-drinking days per week in civilians and veterans with comorbid AUD and PTSD.^28^ Results also showed a trend toward reduced alcohol craving. In the second study, the same dose of prazosin (16 mg/day) was not advantageous over placebo in reducing drinking in veterans with comorbid AUD and PTSD, although drinking did decline over the course of the 12-week study overall.^29^ This study was conducted at two different Veterans Health Administration (VHA) outpatient sites, and alcohol use outcomes were confounded by a site difference, such that better outcomes were demonstrated at the VHA site providing sober housing during treatment. In both studies, prazosin was not more effective than placebo in improving PTSD symptoms or symptom severity.

One other study examined the noradrenergic antidepressant desipramine, a norepinephrine reuptake inhibitor, among veterans with comorbid AUD and PTSD.^19^ In this clinical trial, which did not include a placebo-only control group, desipramine, versus the serotonergic antidepressant paroxetine, decreased the number of drinks per drinking day and the percentage of heavy-drinking days. Like the two prazosin studies, there was a decrease in PTSD symptoms over time but no significant differences between medications.

## Other Noradrenergic Medications

Of the two studies that evaluated prazosin for co-occurring AUD and PTSD, only one found an effect on drinking behavior,^28^ and neither found an effect on PTSD outcomes.^28^,^29^ While this is discouraging, a recent human laboratory study indicated that doxazosin, another alpha_1_-adrenergic antagonist, was effective in reducing alcohol consumption in individuals with AUD who had a strong family history of alcohol problems.^30^ Studies on doxazosin for PTSD also indicate that the drug may be effective in reducing some PTSD symptoms.^31^ Doxazosin is also currently being studied in individuals with comorbid AUD and PTSD. Doxazosin may be more advantageous than prazosin for the treatment of either indication alone, or their comorbidity, due to the long-acting nature of the drug. Doxazosin has a half-life of approximately 18 hours, whereas prazosin has a half-life of approximately 2 to 4 hours. Thus, medication adherence and study retention may improve due to a once-daily dosing schedule of doxazosin compared with multiple prazosin doses throughout the day.

Like prazosin and doxazosin, propranolol also targets the noradrenergic system, but at beta-adrenergic receptors, and it may be a treatment option for individuals with comorbid AUD and PTSD. While limited, studies in humans have shown that propranolol reduces alcohol craving and somatic symptoms associated with alcohol withdrawal,^32^ and previous literature has demonstrated the efficacy of propranolol in reducing intrusive traumatic memories and flashbacks associated with PTSD.^33^

More recently, there has been interest in the ability of propranolol to disrupt drug-related memory reconsolidation, which may be effective in reducing rates of drug relapse. In rodents, repeated propranolol administration disrupted the memory for alcohol-cue associations, such that animals reduced responding for alcohol,^34^ but results have not been consistent.^35^ In humans, propranolol decreased drug craving when administered before memory reactivation through a script detailing a personalized drug-taking experience.^36^ However, like the preclinical findings, studies in humans have had mixed results regarding propranolol’s ability to disrupt drug-associated memory reconsolidation.^37^ Also, to our knowledge, propranolol has not yet been tested specifically in humans for alcohol-associated memories.

Propranolol has also been tested for its ability to disrupt trauma-related memories. Evidence suggests that propranolol effectively reduces physiologic reactivity, fear-rated memories associated with trauma, and PTSD severity, if given soon after a traumatic event,^38^ and it may be used as a strategy to reduce the development or severity of PTSD.^39^ Because propranolol demonstrates efficacy in reducing alcohol-motivated behavior, attenuating PTSD symptoms, and disrupting both drug- and trauma-associated memory reconsolidation, propranolol may also be effective in reducing alcohol use outcomes and PTSD symptom severity in individuals with comorbid AUD and PTSD, providing another potential avenue for treatment and clinical improvement.

## Agents Acting on the Serotonergic System

Selective serotonin reuptake inhibitors (SSRIs) have been the first-line of treatment for PTSD, with only two SSRIs FDA-approved to treat PTSD—sertraline and paroxetine.^40^ However, the efficacy of SSRIs in treating PTSD and associated symptoms is limited, with less than 20% to 30% of patients achieving full remission.^41^ Similarly, findings on SSRIs for the treatment of AUD or associated symptoms are limited.^42^ To date, few studies have examined the effect of SSRIs on comorbid PTSD and AUD conditions. In the 1990s, Brady and colleagues conducted a small open-label pilot study of 200 mg/day of sertraline in individuals with comorbid PTSD and AUD.^43^ Participants self-reported alcohol consumption, and the researchers found that sertraline effectively reduced PTSD symptoms and the average number of drinks consumed, and it increased the number of days of alcohol abstinence. Following these positive preliminary findings, larger trials generally have been less successful at using sertraline to treat alcohol-motivated behavior and have had only modest success using sertraline to treat PTSD.^44^,^45^ In these trials, individuals with comorbid AUD and PTSD demonstrated decreases in drinking behavior, but sertraline was no more effective than placebo at influencing alcohol use outcomes.

Regarding PTSD, Brady and colleagues demonstrated a trend such that sertraline decreased PTSD symptom severity and the cluster symptoms of hyperarousal and intrusion to a greater degree than placebo.^44^ Supporting these findings, Hien and colleagues demonstrated greater reductions in PTSD symptoms at the end of treatment for the sertraline-treated group compared with the placebo group,^45^ and this effect was sustained at 6- and 12-month follow-up. Interestingly, when treated with sertraline, a subgroup of individuals with early-onset PTSD and less severe AUD had more improvement in alcohol use outcomes than individuals treated with sertraline who had late-onset PTSD and more severe AUD.^44^ Further, a subsequent secondary data analysis concluded that improved PTSD symptoms, particularly hyperarousal, were associated with improved alcohol-related symptoms,^46^ possibly suggesting that treatments targeted at reducing hyperarousal or hyperreactivity may be more beneficial in reducing symptoms of both AUD and PTSD in this comorbid population.

Another study examined an FDA-approved medication for the treatment of PTSD in veterans with a dual diagnosis of AUD and PTSD.^19^ Paroxetine was not better than desipramine in reducing percent heavy-drinking days or drinks per drinking day, but paroxetine was comparable to desipramine in reducing PTSD symptoms. As previously discussed, naltrexone in addition to paroxetine or desipramine reduced alcohol craving, but there was no significant additive effect of naltrexone in combination with paroxetine or desipramine on drinking or PTSD symptoms.

Finally, although not an open-label or randomized controlled trial, a retrospective study evaluated the efficacy of quetiapine, an atypical antipsychotic with antagonist effects at serotonin 5-HT_2_ receptors, among veterans with AUD, of whom 90% were diagnosed with PTSD.^47^ These veterans had been treated with quetiapine for sleep disturbances, as older and more recent work has shown that quetiapine is effective in reducing disturbed sleep and other symptoms associated with PTSD.^48^,^49^ This retrospective study aimed to target alcohol use outcomes, thus changes in PTSD symptom severity were not reported. Quetiapine, when compared with placebo, decreased the number of times admitted for detoxification, increased the total number of days abstinent from alcohol use, and trended toward increasing time to relapse. While quetiapine reduced alcohol craving and alcohol consumption in individuals with AUD in preliminary human laboratory, open-label, and retrospective studies, it was not efficacious in reducing drinking outcomes in a large, multisite clinical trial.^50^

## Other Serotonergic Medications

As previously mentioned, sertraline and paroxetine are the only two FDA-approved medications to treat PTSD, and evidence suggests that these medications target PTSD symptom severity, versus the overall reduction or remission of PTSD symptoms, in individuals without AUD and PTSD comorbidity.^51^ Further, based on findings in this review, sertraline yields mixed results in comorbid populations regarding the reduction of alcohol use outcomes and PTSD symptoms. Trazodone, a second-generation antidepressant and antagonist at serotonin 5-HT_2_ and alpha_1_-adrenergic receptors, is prescribed off-label for singular AUD or PTSD and may be an effective second-line treatment for individuals with co-occurring AUD and PTSD. Likely due to trazodone’s anxiolytic- and sedative-like properties, early studies demonstrated that trazodone improved sleep disturbances associated with AUD and alcohol withdrawal.^52^ However, in a study of alcohol detoxification patients, the trazodone-treated group increased alcohol consumption following cessation of the medication.^53^

Regarding PTSD, older studies demonstrated that trazodone decreased PTSD symptoms and dysregulated sleep associated with PTSD.^54^ In individuals with co-occurring substance abuse and anxiety symptoms, including PTSD symptoms, trazodone decreased alcohol consumption and reduced anxiety symptoms.^55^ While trazodone has not yet been investigated in individuals with comorbid AUD and PTSD, and recently published studies on the efficacy of trazodone for either indication remain elusive, there is some evidence suggesting that trazodone may be clinically useful for treating sleep disturbances associated with both AUD and PTSD and possibly their comorbidity. However, results should be interpreted with caution until further studies can establish the safety and efficacy of trazodone in AUD and PTSD populations.

Further, 3,4-methylenedioxy-methamphetamine (MDMA) has shown promise for treatment-resistant and chronic PTSD.^56^,^57^ MDMA, a derivative of methamphetamine, primarily acts to increase the net release of serotonin, although it may stimulate the release of other monoamine neurotransmitters (dopamine and noradrenaline) as well. Pilot studies and a long-term follow-up study demonstrated that MDMA-assisted psychotherapy reduced PTSD symptoms and increased self-reported improvement in individuals with resistant, chronic PTSD.^58^ While these results are encouraging for PTSD, to our knowledge, MDMA has not been investigated as a treatment for AUD or comorbid AUD and PTSD. The abuse liability of MDMA may make it less desirable as a medication for the treatment of any substance use disorder (SUD), including AUD.

## Agents Acting on the GABAergic and Glutamatergic Systems

There is promising evidence suggesting that the GABA and glutamate systems may be targets for treating comorbid AUD and PTSD.^59^ While not FDA-approved for the treatment of AUD, topiramate, an anticonvulsant with action at both GABA and glutamate receptors, has demonstrated efficacy in reducing alcohol consumption in humans and is recommended as a second-line treatment.^10^ Furthermore, other studies suggest that topiramate may be effective in treating PTSD.^60^ Contributing to the framework for studying topiramate in this comorbid population, an 8-week, open-label pilot study assessed the effect of topiramate among veterans with PTSD.^61^ These veterans did not have co-occurring AUD and PTSD, but the authors examined the effect of topiramate on alcohol use and PTSD symptoms. In this study, topiramate was effective in reducing drinking behavior in individuals with high-risk drinking patterns, as well as reducing nightmares and sleep disturbances associated with PTSD. Because the results from this pilot trial and other research demonstrated the efficacy of topiramate for either AUD or PTSD, Batki and colleagues conducted the first randomized controlled trial of topiramate among veterans who have comorbid AUD and PTSD.^62^ Topiramate, when compared with placebo, was effective in decreasing alcohol craving and the percentage of drinking days, and topiramate trended toward decreasing PTSD symptom severity and hyperarousal. It should be noted that there were significant cognitive effects of topiramate on learning and memory in this study, but these cognitive deficits improved by the end of treatment.

## Other GABAergic and Glutamatergic Medications

Zonisamide is an anticonvulsant agent similar to topiramate, but it may have fewer side effects. This may be due to the more indirect effect of zonisamide on GABA and glutamate activity, compared with topiramate.^63^ A small study evaluating the efficacy of zonisamide in the treatment of AUD showed that zonisamide was well-tolerated and reduced heavy-drinking days, drinks per week, and alcohol urges,^63^ and a small pilot study suggests its safety in comorbidity (I. L. Petrakis, personal communication, 2018).

Gabapentin and pregabalin, other FDA-approved anticonvulsants exerting action on GABA synthesis in the brain, have been studied to a moderate extent for their potential in treating AUD and alcohol withdrawal syndrome.^64^ In individuals with AUD, gabapentin effectively reduced heavy drinking and alcohol craving, and it improved rates of abstinence,^65^ although results are mixed, with some findings indicating that gabapentin is more efficacious in individuals with a history of alcohol withdrawal.^66^ Pregabalin is more potent than gabapentin and also has positive effects on alcohol craving and withdrawal.^67^ Because of the anxiolytic properties of both drugs, including their role in reducing generalized anxiety, these agents may hold promise in diminishing symptoms associated with PTSD. Some case reports and retrospective studies confer an advantage of gabapentin over placebo in reducing flashbacks, nightmares, and other sleep disturbances.^68^,^69^ In a randomized controlled trial and case report, pregabalin, when administered in addition to standard medication, also improved PTSD symptom severity, hyperarousal, and sleep disturbances in individuals with combat-related PTSD or sexual trauma.^70^,^71^ While these anticonvulsants have modest efficacy in reducing drinking behavior and PTSD symptoms independently, they should not be ruled out as secondary treatment options for individuals with co-occurring AUD and PTSD who are unresponsive to first-line treatments, especially for individuals who have alcohol withdrawal syndrome or sleep problems associated with PTSD.

Recent evidence also suggests a role for the metabotropic glutamate receptor 5 (mGluR5) in the pathophysiology of PTSD and AUD. Preclinical studies indicate that mGluR5 activity may mediate fear conditioning^72^ and regulate alcohol-related behavior.^73^ Indeed, antagonists at mGluR5 sites, such as 2-methyl-6-(phenylethynyl)-pyridine (MPEP), block the acquisition of fear and decrease alcohol self-administration and reinstatement in rodents.^73^,^74^ In humans, new positron emission tomography (PET) neuroimaging results demonstrate higher mGluR5 availability and positive correlations between mGluR5 availability and avoidance symptoms in individuals with PTSD.^75^ This makes sense, considering that the preclinical literature indicates that mGluR5 receptors are involved in the regulation of fear and stress-related behaviors.^72^ Likewise, hyperactivity at glutamatergic receptors is associated with chronic alcohol misuse,^76^ and PET studies have demonstrated alterations in mGluR5 availability in individuals with AUD, including those who are abstinent.^77^

Taken together, blocking mGluR5 sites may be beneficial in reducing both PTSD-related symptoms and alcohol use outcomes in individuals with both disorders. Although not yet empirically tested, mGluR5 antagonism could provide another new approach for treating comorbid AUD and PTSD. It should be noted that there may be unwanted effects associated with GABAergic or glutamatergic medications, namely cognitive impairment.^62^,^76^ Therefore, treatment approaches involving drugs targeted at the GABA or glutamate neurotransmitter systems may be warranted only in individuals unresponsive to other treatment options.

## Other Targets

Neurokinin-1 receptors have also been targeted as having an effect on alcohol-motivated behavior because of their role in the stress response, with results indicating efficacy in reducing alcohol craving and cortisol reactivity in humans^78^ and in blocking stress-induced reinstatement of alcohol-seeking in rodents.^79^ However, in a human experimental study of individuals with co-occurring AUD and PTSD, aprepitant, a neurokinin-1 receptor antagonist, demonstrated no advantage over placebo in decreasing alcohol craving, subjective responses to stress or alcohol cues, or PTSD symptom severity.^80^

Other treatment targets may include the antioxidant *N*-acetylcysteine, the novel vasopressin 1b receptor antagonist ABT-436, and the neuropeptide oxytocin. A recent pilot trial examined the effect of *N*-acetylcysteine or placebo in veterans with comorbid PTSD and SUD and found *N*-acetylcysteine to be more effective than the placebo in reducing drug craving and PTSD symptoms.^81^ Preclinical work has shown that *N*-acetylcysteine reduced alcohol-seeking and reacquisition of alcohol self-administration in rodents.^82^ Another recent clinical trial examined the effect of ABT-436 in individuals with AUD only and found that ABT-436, when compared with placebo, increased days of abstinence.^83^ Importantly, in a subgroup analysis, individuals with higher baseline levels of stress demonstrated better ABT-436 treatment responses for drinking outcomes. Thus, individuals with AUD and high stress may benefit most from vasopressin 1b antagonism, likely indicating that ABT-436 may be an effective, promising pharmacologic treatment option for individuals with comorbid AUD and PTSD.

Because of its anxiolytic properties,^84^ oxytocin also presents as a potential candidate for the treatment of PTSD^85^ and AUD.^86^ In patients with PTSD, oxytocin decreased total PTSD symptoms provoked by exposure to a traumatic script, the intensity of recurrent thoughts about trauma, subjective anxiety and tension, and amygdala reactivity to emotional faces.^87^ Oxytocin also reduced alcohol withdrawal in patients with AUD,^88^ and it may moderate cue-induced alcohol craving in a subset of individuals who have anxiety and AUD.^89^ To our knowledge, oxytocin has yet to be tested in a comorbid population. These avenues should be explored in future investigations.

## Combination Pharmacotherapies

Combination pharmacotherapy may be another viable treatment option for co-occurring AUD and PTSD, as the clinical efficacy of monotherapy is limited to modest in treating both alcohol use and PTSD symptoms in this comorbid population. In preclinical studies, prazosin, naltrexone, and propranolol all singularly reduced responding for alcohol and decreased alcohol self-administration, but these drugs also reduced other palatable, oral reinforcers.^90^ Subthreshold dosing combinations can be used on the basis that a combination of already efficacious medications can target multiple neural systems. Or, combined medications can target one neural system but affect different receptor subtypes that may be dysregulated in each disorder, thus addressing different symptoms or aspects of behavior. Similarly, medications with different mechanisms of action can be used in combination and in a lower dose range to potentially minimize side effects associated with higher doses of one drug alone, possibly improving medication compliance and study retention.^91^

Work in rodents confirms that combination pharmacotherapy may be a promising treatment approach for AUD. When administered in combination, prazosin and propranolol, two drugs targeting different receptor subtypes within the same neural system, were more effective than either drug alone in decreasing alcohol intake.^90^,^92^ Further, prazosin in combination with naltrexone, two drugs targeting different neural systems, was more effective in reducing alcohol-seeking and consumption than either drug alone.^90^,^93^

This combination approach has also been proposed as a treatment strategy for PTSD to optimize treatment response and prevention.^33^ Medications within the noradrenergic system but with differing mechanisms of action have been shown to treat separate symptoms of PTSD. For example, prazosin, the alpha_1_-adrenergic receptor antagonist, reduces combat-related nightmares and insomnia; whereas propranolol, the beta-adrenergic receptor antagonist, decreases flashbacks and traumatic memories associated with PTSD. As such, Shad and colleagues postulated that prazosin in combination with propranolol may lead to significant clinical improvement of PTSD by treating a broader spectrum of PTSD-related symptoms, an effect not demonstrated with monotherapy.^33^

Further, a fairly recent case report suggests that prazosin in combination with naltrexone was effective in reducing alcohol craving and PTSD-related flashbacks within 4 days of treatment, with complete remission of alcohol craving and flashbacks in 2 to 4 weeks.^94^ It should be noted that these findings were from a single male subject diagnosed with AUD, PTSD, and bipolar II disorder who was taking lithium concurrently with prazosin and naltrexone. To our knowledge, combination pharmacotherapy targeting the noradrenergic system has not yet been tested in human laboratory studies or pilot trials of individuals with co-occurring AUD and PTSD and may be one possible direction to guide optimal and novel clinical treatment approaches for this vulnerable comorbid population.

## Clinical and Research Implications

To date, only 12 studies have examined pharmacologic treatment for co-occurring AUD and PTSD. Three studies targeted mainly the opioidergic system, two targeted the noradrenergic system, four targeted the serotonergic system, two targeted the GABAergic and glutamatergic system, and one targeted the neurokinin-1 receptor. Consistent with conclusions from the recent comprehensive review by Petrakis and Simpson,^16^ there are contradictory findings within each neurobiological system targeted. Overall, findings within the opioidergic system demonstrated a modest reduction in alcohol use outcomes. Prazosin, a target within the noradrenergic system, yielded mixed results regarding alcohol use, and neither of the two studies found an effect on PTSD outcomes. Serotonergic medications also yielded mixed results on alcohol use outcomes but tended to improve PTSD symptoms overall. Topiramate, acting at both GABA and glutamate receptors, reduced drinking behavior and improved PTSD symptoms. While topiramate may stand out as the most promising medication for comorbid AUD and PTSD, larger studies need to be conducted to evaluate its safety and efficacy, especially given the cognitive side effects of the drug. Future work should consider investigating lower doses of topiramate to decrease side effects and improve personalized medicine.^95^

Several factors may contribute to the overall mixed results. Sample sizes were relatively small for half of the studies. While some studies included women, others examined only men or few women. This gender gap could be problematic, as recent research indicates that medication response may differ by gender for naltrexone, some serotonergic medications, and noradrenergic targets. For example, in one study, women’s responsiveness to naltrexone varied across the menstrual cycle, and, during the luteal and early follicular phases, treatment with naltrexone increased serum cortisol,^96^ which may have implications for stress reactivity in both AUD and PTSD. Other research suggests that women have better treatment responses to SSRIs, including sertraline, and have fewer associated adverse events.^97^

Recent evidence also suggests that noradrenergic targets for tobacco dependence may differentially attenuate stress reactivity in women and nicotine-related reinforcement in men.^98^ It is plausible that noradrenergic compounds may also preferentially target gender-sensitive systems for AUD and may be more effective in treating women with posttraumatic stress. Further, recent findings suggest that the prevalence of drinking has increased among women over the past decade,^1^ and women have higher rates of PTSD than men.^3^ Thus, it is important to consider sample size and the ability to detect gender differences in medication response when examining pharmacotherapies for comorbid AUD and PTSD, especially given that many studies were conducted primarily in males.

Another challenge in treating comorbid AUD and PTSD may be related to the type of trauma endured prior to the onset of PTSD. For example, half of the studies examining pharmacotherapy for co-occurring AUD and PTSD reviewed in this article investigated treatment effects in veterans, and many of them had combat-related trauma. The other half examined treatment effects in civilian populations with traumas resulting from childhood experiences, sexual assault, physical assault, witnessing death, and natural disasters. To further complicate treatment, at least one study demonstrated that the severity and order of the development of comorbidity may be related to treatment efficacy. Sertraline was more effective in reducing drinking outcomes in individuals with early-onset PTSD and less severe AUD than in those with late-onset PTSD or more severe AUD.^44^ Thus, further research on personalizing treatment to reflect diagnostic onset and trauma type may be a relevant approach when examining novel targets or strategies for co-occurring AUD and PTSD.

Given the high rates of comorbidity for these two psychiatric disorders, it is somewhat surprising that so few studies have examined effective pharmacologic treatment options. This could be due to the complexity associated with psychiatric comorbidity and the difficulties of conducting research among this population. Treatment studies tend to focus on the effect of medication on one disorder, often excluding for comorbidity. However, real-world clinical populations often include comorbid conditions, further emphasizing the urgent need to examine better pharmacotherapies for improving co-occurring AUD and PTSD in a clinically meaningful way.

Promising targets within each system have demonstrated efficacy in treating independent diagnoses of both AUD and PTSD. For example, nalmefene, doxazosin, propranolol, trazodone, gabapentin, and pregabalin have all been found to reduce alcohol- and PTSD-related outcomes, but they have not yet been tested in comorbid populations. Further, subthreshold combination pharmacotherapy in animal models has been efficacious in reducing alcohol-motivated behavior, and may be an effective strategy for individuals who are unresponsive to first-line treatments or for those who are sensitive to adverse events associated with higher doses of a singular drug.

There is a rich literature on behavioral treatments for comorbid AUD and PTSD that is beyond the scope of the current review.^17^ However, future research should also consider examining behavioral interventions in combination with these novel pharmacotherapies to better manage alcohol use outcomes and PTSD symptoms in this comorbid population. Human laboratory studies provide an efficient, cost-effective avenue for evaluating the effects of potential medications on psychiatric disorders. This method has been used effectively to screen medications for drug use disorders.^99^ When examining treatments for co-occurring AUD and PTSD, investigators are encouraged to use promising treatment targets or their combinations. Also, researchers can use human laboratory paradigms to screen these potential medications in an effort to optimize the clinical utility of pharmacotherapeutic treatments for comorbid AUD and PTSD.

## Conclusion

Pharmacotherapeutic treatment options for co-occurring AUD and PTSD are limited. To date, only 12 studies have examined pharmacologic interventions in this comorbid population, and most demonstrated only modest efficacy, but results are mixed. While not comprehensive of all neurobiological systems that may be dysregulated by alcohol use and post-traumatic stress, the existing literature has focused on the opioidergic, noradrenergic, serotonergic, and GABAergic/ glutamatergic systems. Targeting other promising, efficacious medications within these neurobiological systems, or combining medications within the same system or across systems, may be an important and promising next step in treating comorbid AUD and PTSD, especially among individuals who are unresponsive to first-line treatments. Future studies need to urgently address this critical literature gap in order to advance pharmacotherapeutic treatment options in special populations with co-occurring AUD and PTSD.
